# Truncation effect reduction for fast iterative reconstruction in cone-beam CT

**DOI:** 10.1186/s12880-022-00881-8

**Published:** 2022-09-05

**Authors:** Sorapong Aootaphao, Saowapak S. Thongvigitmanee, Puttisak Puttawibul, Pairash Thajchayapong

**Affiliations:** 1 Faculty of Medicine, Prince of Songkla University, Songkhla, Thailand; 2 Medical Imaging System Research Team, Assistive Technology and Medical Devices Research Center, National Science and Technology Development Agency, Pathum Thani, Thailand; 3 National Science and Technology Development Agency, Pathum Thani, Thailand

**Keywords:** Cone-beam CT, Iterative reconstruction, Scattering radiation, Metal artifact, Truncation artefact

## Abstract

**Background:**

Iterative reconstruction for cone-beam computed tomography (CBCT) has been applied to improve image quality and reduce radiation dose. In a case where an object’s actual projection is larger than a flat panel detector, CBCT images contain truncated data or incomplete projections, which degrade image quality inside the field of view (FOV). In this work, we propose truncation effect reduction for fast iterative reconstruction in CBCT imaging.

**Methods:**

The volume matrix size of the FOV and the height of projection images were extrapolated to a suitable size. These extended projections were reconstructed by fast iterative reconstruction. Moreover, a smoothing parameter for noise regularization in iterative reconstruction was modified to reduce the accumulated error while processing. The proposed work was evaluated by image quality measurements and compared with conventional filtered backprojection (FBP). To validate the proposed method, we used a head phantom for evaluation and preliminarily tested on a human dataset.

**Results:**

In the experimental results, the reconstructed images from the head phantom showed enhanced image quality. In addition, fast iterative reconstruction can be run continuously while maintaining a consistent mean-percentage-error value for many iterations. The contrast-to-noise ratio of the soft-tissue images was improved. Visualization of low contrast in the ventricle and soft-tissue images was much improved compared to those from FBP using the same dose index of 5 mGy.

**Conclusions:**

Our proposed method showed satisfactory performance to reduce the truncation effect, especially inside the FOV with better image quality for soft-tissue imaging. The convergence of fast iterative reconstruction tends to be stable for many iterations.

## Background

Cone-beam computed tomography (CBCT) technology, which uses an X-ray source and a flat-panel detector (FPD), is widely used due to its small machine size, large field of view (FOV), and low radiation dose [1–3]. Most of this technology is applied for diagnosis and treatment planning, such as dentomaxillofacial, orthopaedic, and breast-cancer specimen imaging [4, 5]. The quality of images reconstructed from CBCT is often deteriorated by many causes, such as X-ray scattering, beam hardening, metal artifacts, and truncation artifacts [4, 6–11]. Specifically, truncated data or incomplete projection images are often noticeable when an object is larger than a detector active area and thus directly affecting image quality and reconstruction. As a result, hard-tissue and soft-tissue imaging cannot be clearly visualized in the reconstructed images. However, good image quality in CBCT imaging is still required and important for diagnosis of the diseases. Generally, artifacts from the truncation effects are easy to handle with filtered back projection (FBP) reconstruction but not iterative reconstruction (IR). Compared to FBP, IR is more susceptible to incomplete data due to accumulation of projection errors in each iteration of projection.

Many researchers have studied and developed truncation effect reduction in CBCT imaging. Ohnesorge et al. [6] estimated the lost data in projection images using symmetric mirroring extrapolation, and Hsieh et al. [7] approximated the lost data with extrapolation using a fitted water cylinder. Both techniques extended projection images and synthesized new information that filled truncated projection images during the convolution process in FBP. Maltz et al. [12] proposed CT truncation artifact removal using water-equivalent thickness. This method assumed that the outline of large body parts could be roughly approximated as an ellipse to predict and estimate the lost data in the truncated or incomplete projection images. Another research published by Dang et al. [13] attempted to mitigate the truncation effect in iterative reconstruction by a downsampling method and an extension of the FOV, but that was applied only on the extended FOV in the axial plane of the reconstructed image. In fact, the truncation artifact can appear not only on the axial plane but also on the sagittal and coronal planes of the FOV, i.e., the upper and lower parts of the head.

Herein, we propose a novel truncation reduction method for CBCT. Our proposed method expands the size of the FOV and extrapolates the height of projection images including an image smoothing parameter for noise regularization in reconstruction. Fast iterative reconstruction is employed using the modified convex algorithm with an additional penalized likelihood estimation called the PL-C algorithm [14–18]. Then, the PL-C is additionally modified to accelerate the convergence by using acceleration techniques [19–21]. To validate the proposed work, the method was experimentally validated with an anthropomorphic head phantom and a real human head. The validation results were compared against those from FBP [22, 23].

## Methods

**Fig. 1:**
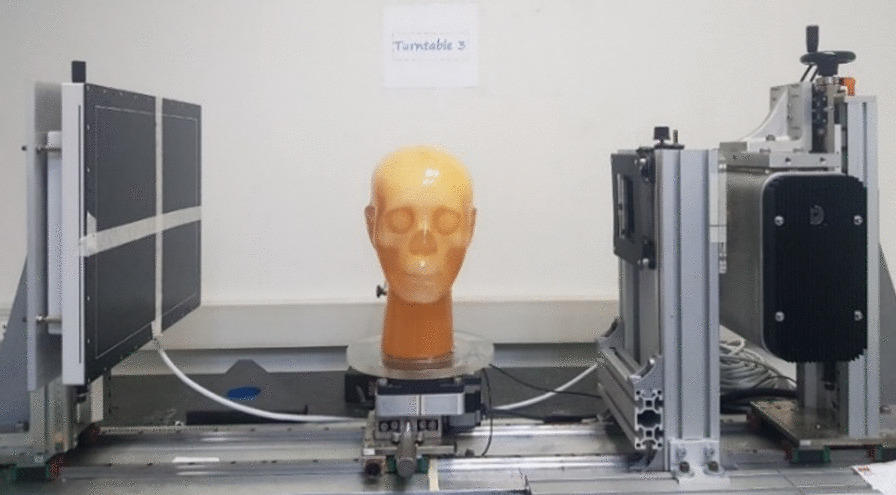
The CBCT scanner prototype on a benchtop system

A CBCT scanner prototype was installed on a benchtop system as shown in Fig. 1. The system consisted of an X-ray source, a rotation stage using a stepping motor, and a flat panel detector based on amorphous silicon thin-film transistors. The X-ray source has a maximum power of 5 kW; tube potential of 50–120 kV, a focal spot size of 0.6 mm; a rotating anode, and a 15° target angle. The detector has a size of 40 cm × 30 cm and a pixel pitch of 0.194 mm. The distance from source to detector (DSD) in this system is 950 mm and the distance from source to object (DSO) is 500 mm. The center of the X-ray is aligned toward the center of the flat panel detector (FPD) and the FOV. The projection dataset was acquired using 100 kV, 7 mA, and 40.32 mAs. The computed tomography dose index (CTDI) value used in this system was 5 mGy.

An anthropomorphic head phantom was used for verification of the algorithm and image quality (PH3 angiographic CT head phantom, Kyoto Kagaku, Japan). Moreover, we analyzed the proposed work with one anonymous human head dataset, which was retrospective and used for different purposes. In this case, the human dataset was acquired from our in-house CBCT machine where the gantry was rotated. All of the proposed work used the same computer (Core-i7-9700 CPU at 3.0 GHz, 16 GB RAM) for developing the algorithm and was implemented on a graphic processing unit (GPU) card (GeForce RTX2070, NVIDIA) with a CUDA library.

**Fig. 2:**
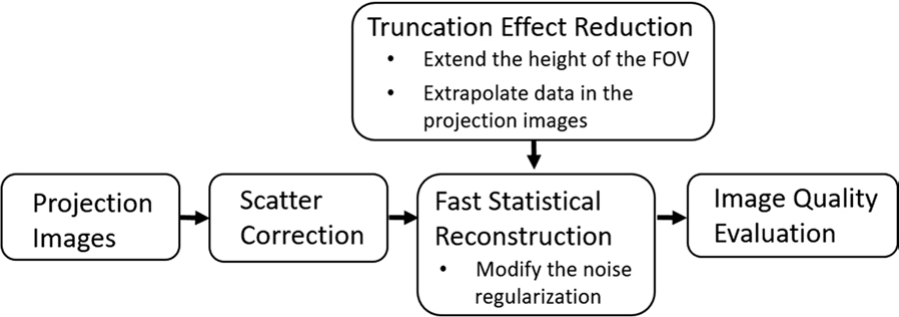
The overall process of the proposed reconstruction

An anthropomorphic head phantom was scanned by the CBCT scanner prototype. In CBCT imaging, we can perform a few processes sequentially, such as scatter correction, fast iterative reconstruction with the proposed truncation effect reduction, and image quality evaluation. These processes can be described in Fig. 2. The in-house scatter correction algorithm [24, 25] was applied to projection data before reconstruction to accurately estimate X-ray scatters.

### Fast iterative reconstruction

Statistical iterative reconstruction is based on maximum likelihood estimation with the convex function [15–18]. The maximum likelihood function $L\left( \mu \right)$ estimates the linear attenuation coefficient in 3D cross-section or volume images from projection images. The objective function of the maximum likelihood algorithm is modified by adding a penalty function to regularize noise. Thus, the estimate of the attenuation is obtained by maximizing the objective function as follows:

1 $$\hat{\mu } = \arg \mathop {\max }\limits_{\mu \ge 0} {\Phi }\left( \mu \right)$$

where the objection function ${\Phi }$ is given by

2 $${\Phi }\left( \mu \right) = L\left( \mu \right) - \beta R\left( \mu \right)$$

where $\beta$ is a parameter for smoothness control, $R\left( \mu \right)$ is the roughness penalty based on a potential function ${\uppsi }$ that uses the Huber function [14, 17] for edge preservation:

$${\uppsi } = \left\{ {\begin{array}{*{20}c} {\frac{{\mu^{2} }}{2},} & {\left| \mu \right| \le \delta } \\ {\left| \mu \right| - \frac{{\delta^{2} }}{2},} & {\left| \mu \right| > \delta } \\ \end{array} } \right.$$

where $\delta$ controls the level of edge preservation. The PL-C algorithm [17, 18] is used to calculate the attenuation coefficient in Eq. (1). Thus, the update $\Delta$ of the attenuation coefficient $\mu_{j}$ at iteration *n* + 1 is calculated as follows:

$$\mu_{j}^{n + 1} = \mu_{j}^{n} + {\Delta }$$

Iterative reconstruction is well known for taking a long processing time. Thus, several approaches have been investigated to reduce the processing time. One conventional acceleration technique is to divide the projection images into ordered subsets (OS) [16, 17] to improve the reconstruction speed. Each ordered subset of projections is performed in subiterations for reconstruction. All subiterations are combined to one iteration to update the volume. Therefore, the number of forward and backward operations per update in each iteration is reduced. Yet, the convergence is still slow. Another acceleration technique proposed by Kim and Fessler [19], and Kim et al. [20], modified the momentum between the last and current updates to achieve a faster convergence. Such a method is known as Nesterov (NES) [21] acceleration, which is shown in the equation below:

3 $$\mu_{j}^{n + 1} = \left( {1 - \frac{1}{t}} \right)z + \frac{1}{t}\left( {\mu^{0} - v} \right)/2$$

where

$$z = \mu_{j}^{n} + {\Delta }$$

$$v = v + t \cdot {\Delta }$$

$$t = \left( {1 + \sqrt {1 + 4t^{2} } } \right)/2$$

where *n* is the number of iterations, *z* is the current image estimation, *v* is the summation of the momentum from all subiterations, and *t* is the momentum weight that linearly increases in each subiteration. The parameter of $\mu^{0}$ can be initialized with arbitrary values or FBP. In this work, iterative reconstruction with both acceleration techniques was performed for *n* iterations and *m* subsets denoted by OS-*m* and NES-*m*, respectively.

### Truncation effect reduction

**Fig. 3:**
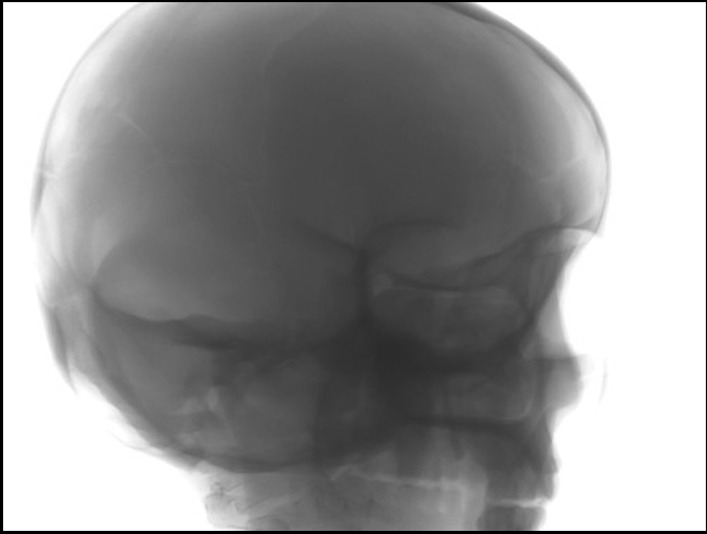
An example of truncation data providing an incomplete projection image in the longitudinal areas of a head phantom

Incomplete projection images or truncated data are often observed while scanning an object that is larger than the detector area. Some data on the peripheral sides may not appear in some projections. In addition, the longitudinal areas of a real human head (along the upper and lower parts) or a head phantom in projection images are usually truncated in most cases as shown in Fig. 3. Thus, truncated data around projection images are still the main cause of the degraded image quality, as it generates new artifacts and directly affects 3D reconstructed images.

**Fig. 4:**
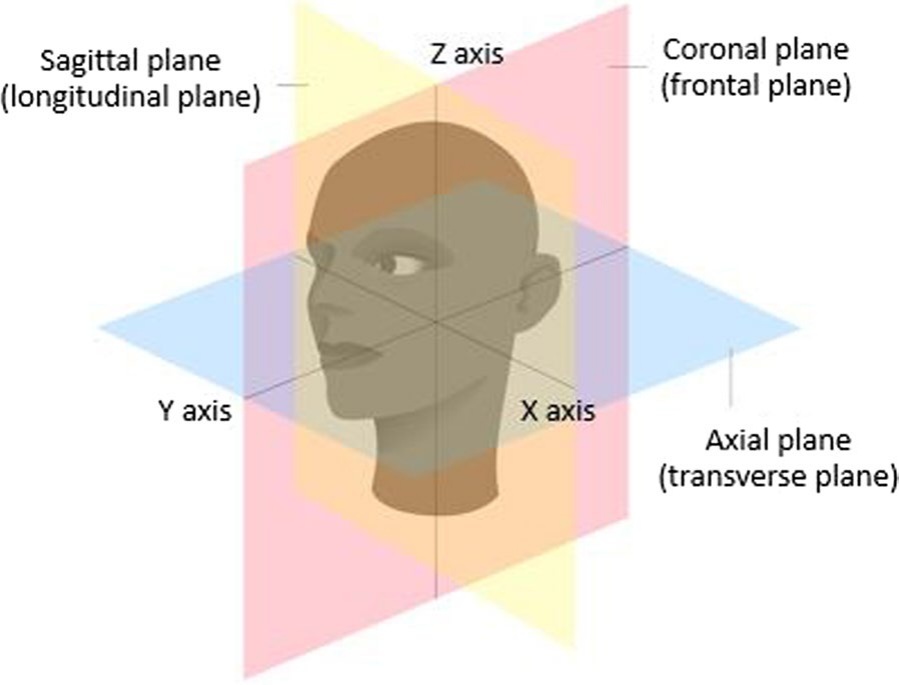
Definition of imaging planes in the volume matrix of the FOV

**Fig. 5:**
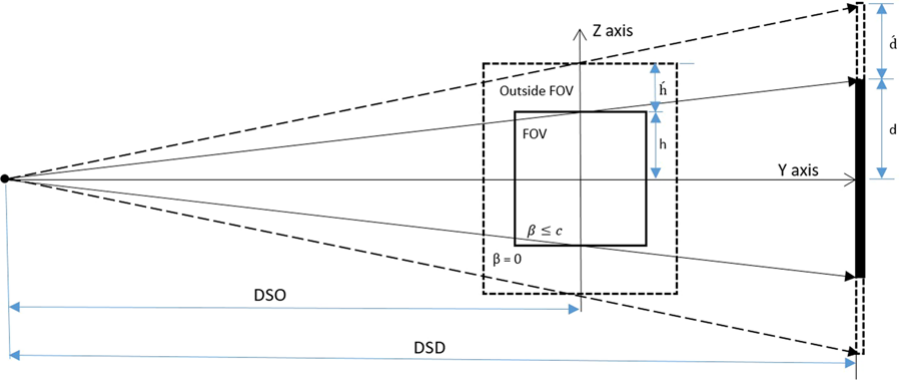
The extended FOV in the Y–Z plane of the volume matrix and the height of the detector

**Fig. 6:**
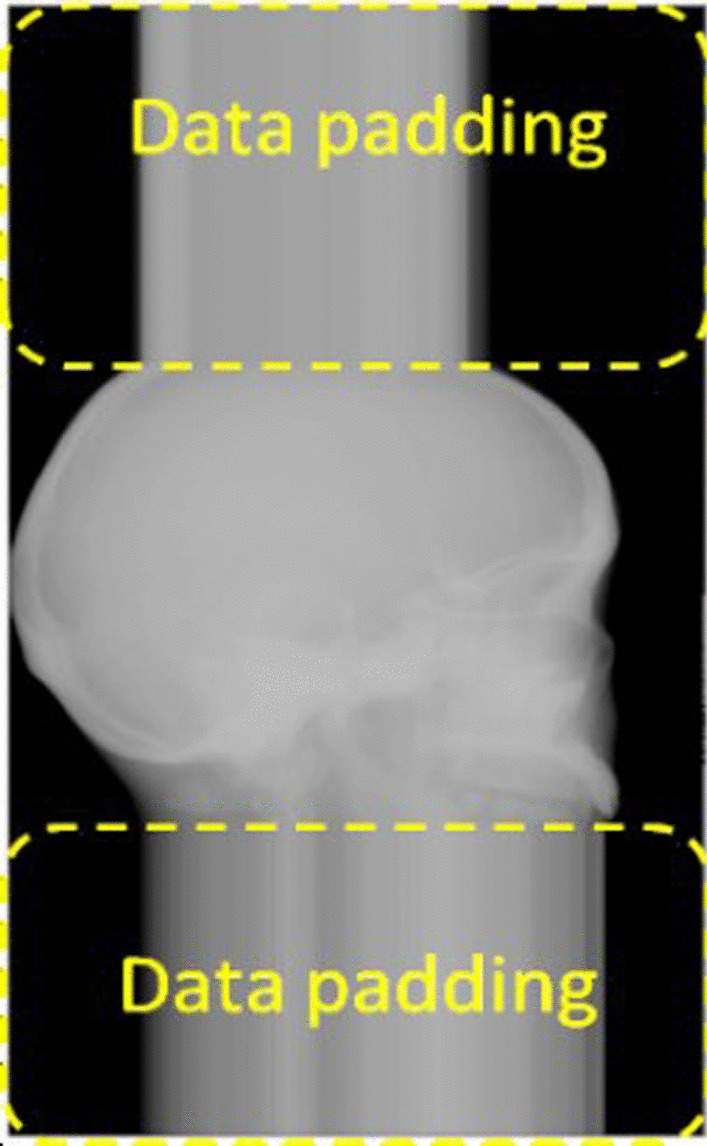
Data extrapolation along the longitudinal plane in the projection image

A conventional technique for truncation effect mitigation in iterative reconstruction is to compensate error data causing large differences between incomplete measurements and calculated forward projections. The compensation extends the matrix volume size of the FOV in the X–Y or axial plane as shown in Fig. 4. However, this technique is insufficient due to the remaining accumulated error in the regions of the Y–Z or longitudinal plane [13] in the FOV. Therefore, in this paper, we propose the extension of the matrix volume size, especially in the longitudinal plane outside the FOV as shown in Fig. 5. Since the extended height of FOV and projection is based on cone-beam geometry, the projection images are modified by extrapolating along the longitudinal planes with the extended height $\acute{d}$ calculated as follows:

4 $$\acute{d} = \left( {h + \acute{h} } \right)\frac{DSD}{{DSO}} - d$$

where *d* is the height of the detector, *h* is the height of the FOV, and $\acute{h}$ is the extended height of the FOV. The variation of $\acute{h}$ affecting the image quality will be studied and discussed. Due to difficulty in simulating the actual information lost in truncated data, we selected the most probable data to use in $\acute{d}$ as data rows at the border of projection images. Therefore, the projection images were extrapolated by the last data rows as shown in Fig. 6. Moreover, we found that a smoothing parameter $\beta$ in the iterative reconstruction could well tackle noise in the reconstructed images, but a large smoothing value may rapidly increase the accumulated error [19, 21, 26].

The accumulated error in each iteration is controlled by limiting $\beta$ to not more than an expected constant value *c* based on acceptable image quality. Thus, the entire extended FOV and projection are performed with fast iteration reconstruction, and we define the reconstruction with different $\beta$ values outside the FOV. The smoothing parameter $\beta$ outside the FOV is set to zero to avoid accumulated error while processing.

### Image quality evaluation

The head phantom was placed partially inside the FOV to simulate the truncation effect. The truncated data or incomplete projection images of the head phantom were acquired from the prototype. They were corrected for X-ray scattering [24, 25] and reconstructed by fast iterative reconstruction with the proposed truncation effect reduction to obtain the reconstructed images. In this case, we focused on certain slices of reconstructed images in the upper part inside the FOV because they tend to degrade easier than other slices. Noise was measured at the slices inside the FOV with different matrix volume sizes in the Y–Z plane. In the performance measurements of convergence in iterative reconstruction with and without the proposed effect reduction, the mean-percentage-error (MPE) value was used inside the region of interest (ROI) in each iteration and normalized by the reconstruction image from OS-1 at 1000 iterations as follows:

5 $$\% MPE = \frac{100}{N} \times \sum \left( {\frac{{\left| {\mu_{OS - 1}^{1000} - \mu^{n} } \right|}}{{\mu_{OS - 1}^{1000} }}} \right)$$

where N is the total number of pixels inside the ROI, *n* is the number of iterations. Moreover, the contrast-to-noise ratio (CNR) value was used for image quality evaluation and comparison with FBP as follows:

6 $$CNR = \frac{{M_{x} - M_{b} }}{{\sqrt {\sigma_{x}^{2} - \sigma_{b}^{2} } }}$$

where $M_{x}$ is the average intensity in the ROI in the reconstructed images, $M_{b}$ is the average intensity of the background, and $\sigma_{x}$ and $\sigma_{b}$ are the standard deviation of the ROI and background, respectively.

## Results

### The truncation effect reduction

**Fig. 7:**
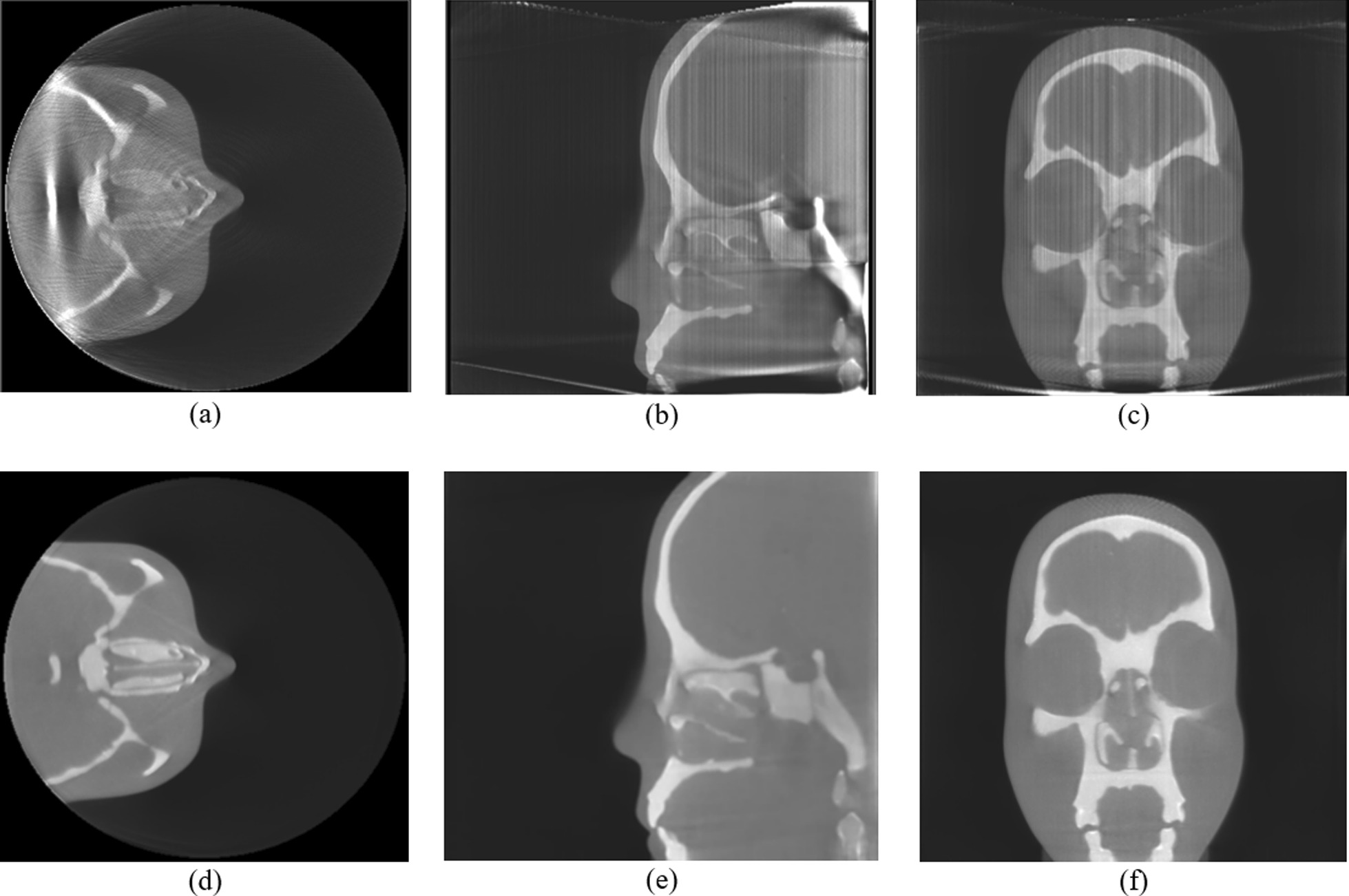
The reconstructed images (1st row) without and (2nd row) with the proposed method using the anamorphic head phantom in **a** and **d** the axial planes, **b** and **e** the sagittal planes, and **c** and **f** the coronal planes, respectively

**Fig. 8:**
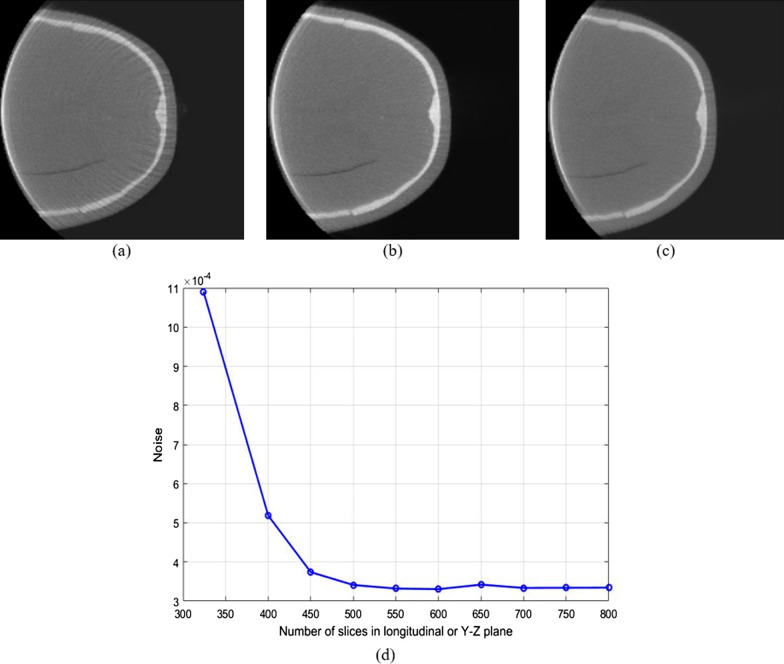
The reconstructed images in the FOV **a** without extended height using 324 slices, **b** with extended height using 450 slices, **c** with extended height using 800 slices. **d** Noise against different numbers of slices in the extended FOV

Figure 7 shows the results of the reconstructed images in the axial, sagittal and coronal planes with and without the proposed work. The PL-C algorithm with the accelerated techniques was applied for the reconstruction using $\beta$ = 150, ${\updelta }$ = 0.00005, OS-10, and 20 iterations. The expected FOV has the volume matrix size of 400 × 400 × 324 voxels while the extended size contained 600 × 600 × 800 voxels with a voxel size of 0.5 mm. In Fig. 7a–c, the reconstructed images without the proposed method showed many shades and streaks from the truncation effect as appeared in the entire reconstructed images of the axial, sagittal, and coronal planes. The results of the reconstructed image with the proposed method are shown in Fig. 7d–f. The artifacts from truncation effects inside the FOV are apparently reduced although they still exist outside the FOV. Figure 8 shows the reconstructed images and the noise measurement with different extended sizes. In the experiment, the image size in the axial or X–Y plane was fixed at 600 × 600 voxels and the number of slices in the longitudinal or Y–Z plane was varied between 324 and 800 slices. The artifacts appeared in the slices are decreased when a number of slices are increased as shown in Fig. 8a–c.

In addition, noise from artifacts is continuously decreased until the algorithm is converged and consistent with the increased height of the FOV as shown in Fig. 8d. Hence, the consistent decreased noise can be the stopping criteria for the extended height of the FOV.

### Mean-percentage-error evaluation

**Fig. 9:**
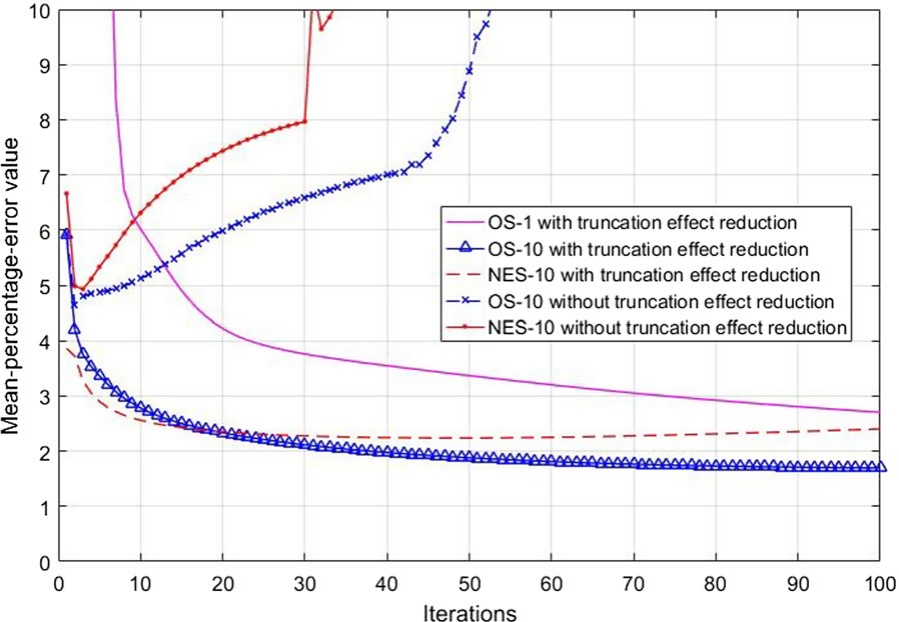
The MPE values using acceleration techniques against each iteration

The MPE value was calculated from the reconstructed images in each iteration using Eq. *(**5**)*. Reconstruction with and without the proposed work was fixed at $\beta$ = 150, $\delta$ = 0.00001, 1000 iterations and varied acceleration techniques as follows: OS-10 and NES-10. The results of MPE were plotted together in Fig. 9. We found that the MPE values of OS-10 and NES-10 without the reduction of truncation effects did not converge but kept increasing. On the contrary, the MPE values of both acceleration methods with the reduction of truncation effects showed a stable convergence at 100 iterations. Though, NES-10 seemed diverging a little after a certain iteration.

### A preliminary result with a human dataset

**Fig. 10:**
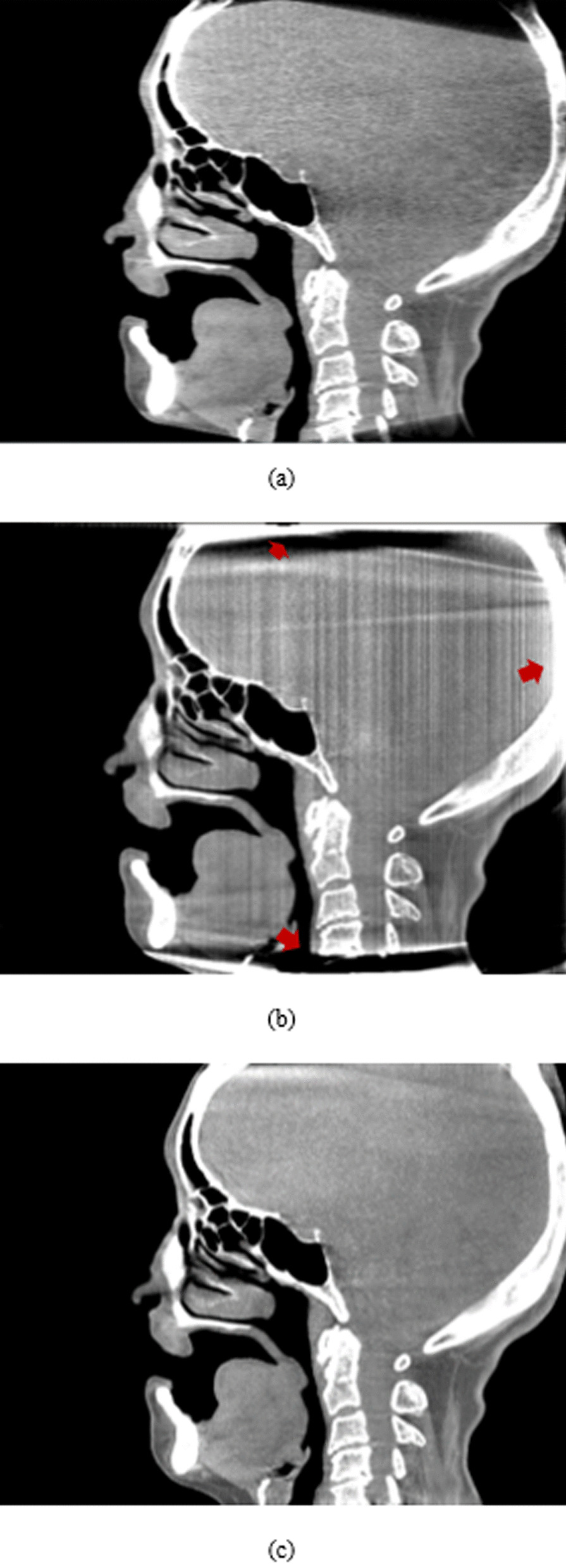
The comparison of a human head dataset in the sagittal planes between **a** FBP, **b** PL-C without truncation effect reduction where the red arrows indicated the artifacts at the truncated border of the image, and **c** PL-C with truncation effect reduction

**Fig. 11:**
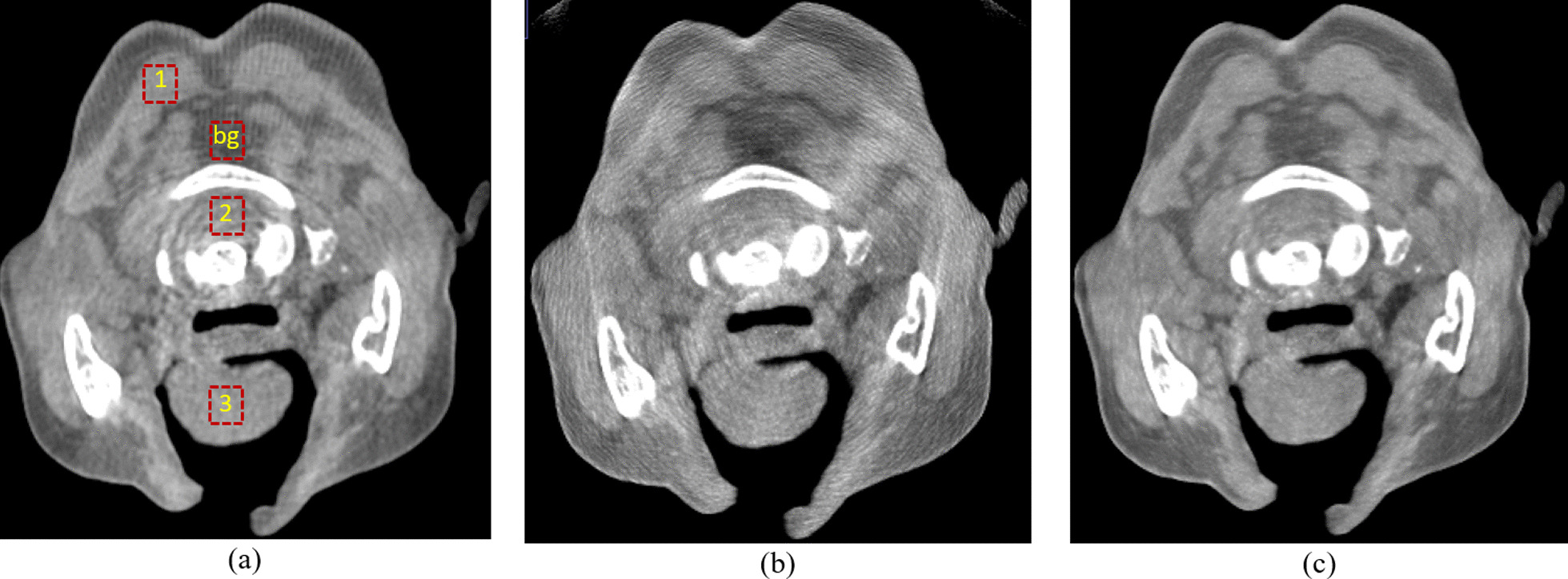
The comparison of soft-tissue images between **a** FBP, **b** PL-C without truncation effect reduction, and **c** PL-C with truncation effect reduction. Note that the CNR measurements were calculated at 3 red squared ROIs against the background

**Fig. 12:**
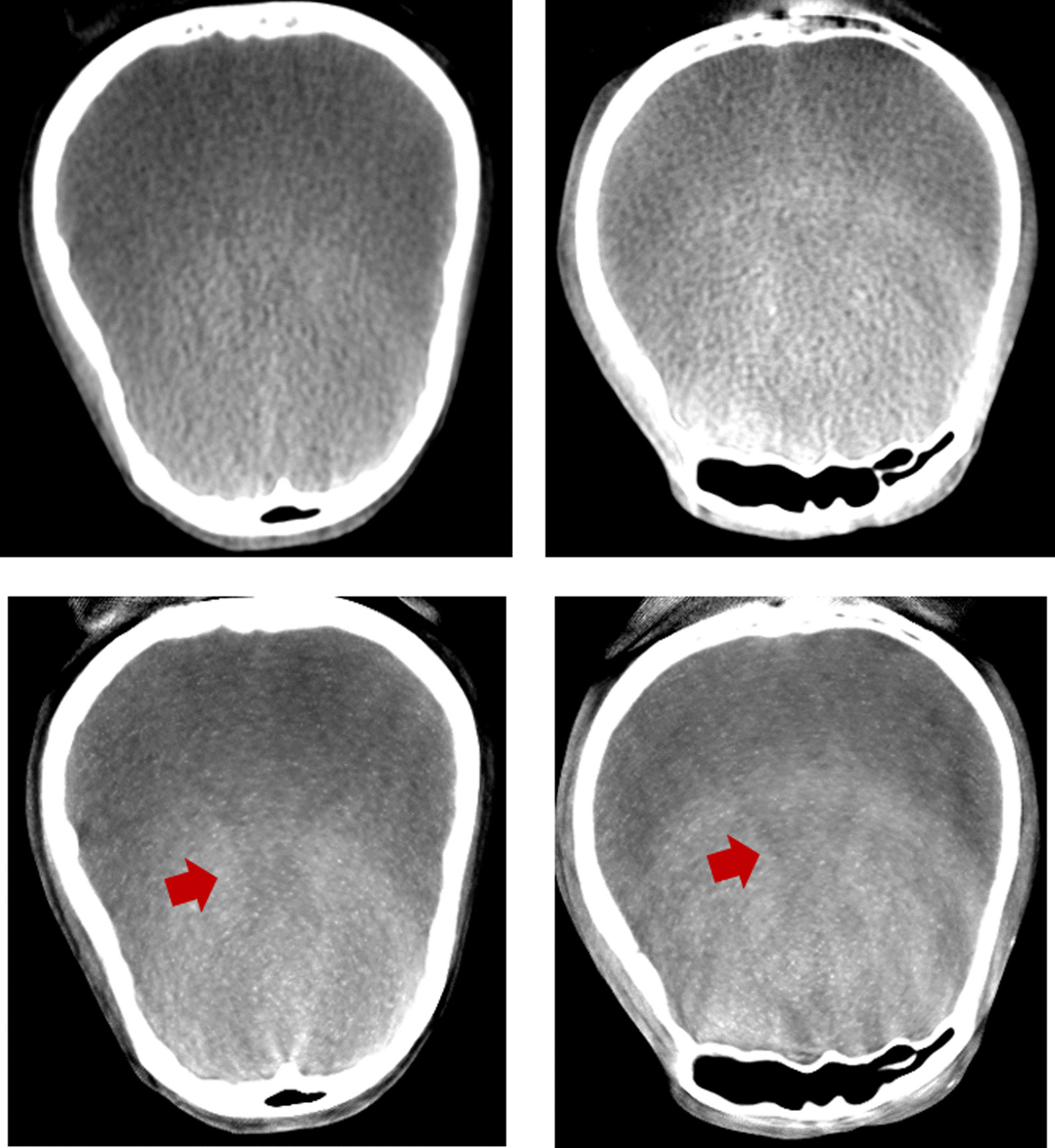
The ventricle-tissue images from (1st row) FBP and (2nd row) the proposed method. Note that the ventricles are indicated by the red arrows

In the experimental results, one real human head dataset using CTDI of 5 mGy was tested with the proposed method. Figure 10 shows a comparison of the sagittal plane results of a human head between the FBP (using the Hamming filter and a cut-off frequency of 0.65, 480 × 480 × 368 voxels) as shown in Fig. 10a and the PL-C algorithm (using $\beta$ = 150, ${\updelta }$ = 0.00001, OS-10, 20 iterations, 600 × 600 × 800 voxels) with and without the proposed truncation effect reduction. In the results of the PL-C algorithm without the truncation effect reduction, the red arrows indicated the artifacts around the truncated border of the images as shown in Fig. 10b. Using the proposed method, the artifacts in these areas could be reduced as shown in Fig. 10c. Moreover, Fig. 11 shows the soft-tissue image results as compared with FBP. The CNR value on soft-tissue images with a ROI size of 20 × 20 pixels was evaluated against the background. The CNR from the PL-C algorithm with the proposed truncation effect reduction is significantly higher than that from others, as shown in Table 1. The processing times of the FBP and PL-C algorithms were 10 s and 4 min, respectively, but the PL-C algorithm with the truncation effect reduction used a longer processing time of 6 min. Moreover, we selected some slices of the ventricle tissue images for comparison between FBP and PL-C with the proposed method were shown in Fig. 12. The ventricle in both brain images from the proposed method can be observed easier than that from FBP apparently as the red arrows indicated in the reconstructed images as shown in the second row of Fig. 12.

**Table 1:** Contrast to noise ratio comparison in the soft-tissue areas

	FBP	PL-C	PL-C with the proposed method
ROI#1	12	13	58
ROI#2	6	10	10
ROI#3	16	9	32

**Fig. 13:**
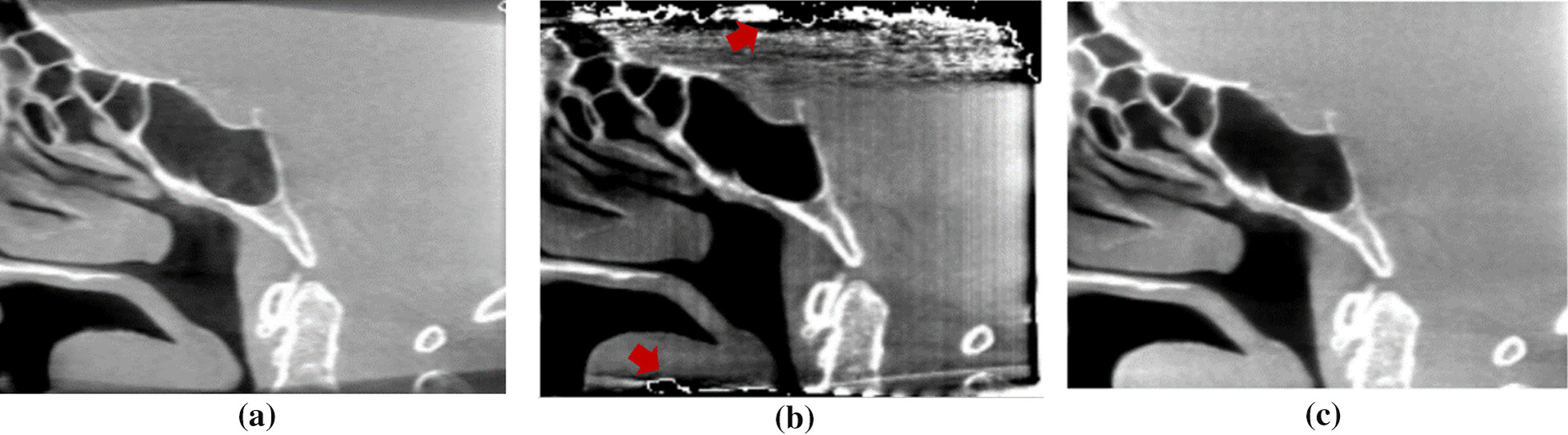
The comparison on a smaller FOV of human head data between **a** FBP, **b** PL-C without truncation effect reduction where the red arrows indicated the zone of artifacts at the truncated border of the image, and **c** PL-C with truncation effect reduction

In addition, to verify the performance of the proposed method, we created another dataset by cropping the previous projection images of the human head to reconstruct the smaller FOV to yield more truncation artifacts. The proposed method still handles the artifacts reasonably well in the reconstructed images when compared to those without truncation effect reduction as shown in Fig. 13.

## Discussion

Truncated data or incomplete projection images are found when an object is larger than a detector active area. The truncation artifacts are highly sensitive to iterative reconstruction. In this work, the size of FOV and the height of projection images were expanded using extrapolation and fast iterative reconstruction was performed using the modified smoothing parameter to reduce the accumulated error. As seen in the reconstructed image of the head phantom and human head, the artifacts inside the FOV are effectively reduced. However, some artifacts are still noticeable outside the FOV because of inaccurate extrapolation and insufficient compensation in reconstruction. The smoothing parameter $\beta$ helps the reduction of noise. Since the noise regularization using the smoothing parameter outside the FOV in the extended areas is out of interest, the penalty term in IR for noise reduction in Eq. (2) is ignored by letting $\beta$ be zero. The accumulated error of the reconstruction is considerably reduced. However, the accumulated error at the high iteration do not decrease because of no weight in the update term of IR, i.e., the weight value helps the compensation of increasing the error at each iteration of reconstruction.

Although the extended height of the FOV can well reduce the accumulated error on the longitudinal plane, the capability of compensation still depends on accurate extrapolation in the extended height area of the projection images. Generally, accurate extrapolation is difficult to synthesize the lost data in the projection images. Although many techniques have attempted to simulate and estimate the lost data in the projections, the errors in the projection images still remain and are increased when fast iterative reconstruction has been run for many iterations. The error from inaccurate extrapolation is expanded from outside to inside the FOV, and that causes the MPE to diverge. Hence, the extended height of the FOV and projections help to mitigate the accumulated error expanding into the areas inside the FOV. For instance, the artifacts in the longitudinal plane inside the entire FOV with the proposed method can be enhanced better than those of the extension method only in the axial plane [13] as the noise is reduced as shown in Fig. 8d.

The acceleration techniques have been implemented in iterative reconstruction to reduce the reconstruction time by speeding up the convergence. However, such techniques have a serious effect on the accumulated error. The faster the convergence of iterative reconstruction is accelerated, the more the accumulated error in the reconstructed images is increased. The OS-10 and NES-10, which are the commonly used acceleration techniques in iterative reconstruction, were evaluated for the accumulated error after applying the proposed truncation effect reduction. Both techniques with the proposed method show decreasing in the accumulated error, while those without the proposed method show the increasing errors. The proposed truncation effect reduction compensates a large difference between the incomplete measurement and calculated forward projection as the extension of the entire FOV and noise regularization outside the FOV. However, the MPE of NES-10 diverges before OS-10 does. Divergence of the MPE is caused by the rapid accumulated errors from many subiterations and momentum weights [21]. The reconstructed images using NES in Eq. (3) are performed by the weight and summation of the momentum to achieve fast convergence, but the error while processing is always accumulated. In addition, increasing a number of subsets on both OS and NES also affects the rapid divergence [21]. Therefore, image quality evaluation and parameters of acceleration techniques in fast iterative reconstruction should be considered together for suitable solutions.

In low radiation dose acquisition, the soft tissues with low contrast often pose a challenge in discrimination of details in a noisy image. The ability of the proposed method to mitigate the noise substantially benefits the visualization images of soft tissues, such as the brain. The image of the brain reconstructed from the proposed method is clearly visualized and easily discriminable. The CNR is greatly improved due to significant noise reduction. The proposed method is evaluated for the image quality of the ventricles, which are the chambers containing the cerebrospinal fluid in the brain and are critical for diagnosis of hydrocephalus and intraventricular hemorrhage [27]. The visualization of the ventricles is challenging in CBCT because of a relatively low contrast of the area. Interestingly, the ventricle images from the proposed method can be observed much clearer compared to those from FBP having the same radiation dose. Here, the radiation dose (CTDI) of the CBCT data is only 5 mGy as compared to 70 mGy of a conventional fan-beam CT.

Even though our proposed method uses fast iterative reconstruction, the processing time of PL-C algorithm is still increased when the truncation effect reduction has been applied. The expected processing time of the PL-C algorithm with the proposed method is optimized by varying the parameters of the acceleration technique including the number of iterations, and the size of extended FOV based on acceptable image quality as 6 min with the proposed method using OS-10 and 20 iterations. Although another study [13] handled the processing time better than the proposed method, it did not mitigate the artifacts in the longitudinal plane of the FOV. The proposed method is able to compromise both image quality and processing time. Nevertheless, the proposed method can achieve good image quality in the reconstructed images inside the entire FOV in exchange for a considerable amount of the computational resources, including the GPU memory and processing time. The performance of the proposed method can be improved by finding an advanced technique to iterative reconstruction to reduce the accumulated error outside the FOV, so that the extension of the FOV can be handled and the GPU memory and processing time can be reduced.

## Conclusions

In this work, we proposed truncation effect reduction for fast iterative reconstruction in CBCT imaging due to truncated data or incomplete projection images acquired from a CBCT scanner. Our proposed work extended the size of the FOV and the height of projection images, including the modified smoothness parameter $\beta$ to zero outside the FOV for reconstruction. Here, fast iterative reconstruction used the PL-C algorithm with the acceleration techniques of the ordered subset and Nesterov’s momentum weight techniques. The results of the head phantom and the human head data from the proposed method showed the significant decrease of the artifacts and the improvements on achieved image quality. The CNR of the soft-tissue images with the proposed method was distinctly improved by the increased contrast and the decreased noise. Visualization of the ventricle and soft-tissue images from the proposed method can be easily observed even with the very low radiation dose. Therefore, our proposed work has satisfactory performance to reduce the truncation effect in the CBCT reconstructed images and enhance soft-tissue imaging.

## Data Availability

The datasets generated and/or analysed during the current study are not publicly available, but are available from the corresponding author on reasonable request.

## Abbreviations

CBCT: Cone-beam computed tomography
FPD: Flat-panel detector
FOV: Field of view
FBP: Filtered backprojection
IR: Iterative reconstruction
PL-C: Modified convex algorithm with additional penalized likelihood estimation
DSD: Distance from source to detector
DSO: Distance from source to object
CTDI: Computed tomography dose index
GPU: Graphic processing unit
OS: Ordered subsets
NES: Nesterov
MPE: Mean-percentage-error
ROI: Region of interest
CNR: Contrast-to-noise ratio
